# Age-Related Impairment of Bones' Adaptive Response to Loading in Mice Is Associated With Sex-Related Deficiencies in Osteoblasts but No Change in Osteocytes†

**DOI:** 10.1002/jbmr.2222

**Published:** 2014-07-21

**Authors:** Lee B Meakin, Gabriel L Galea, Toshihiro Sugiyama, Lance E Lanyon, Joanna S Price

**Affiliations:** School of Veterinary Science, University of BristolLangford, Bristol, UK

**Keywords:** AGING, OSTEOBLASTS, MECHANICAL LOADING

## Abstract

Bones adjust their mass and architecture to be sufficiently robust to withstand functional loading by adapting to their strain environment. This mechanism appears less effective with age, resulting in low bone mass. In male and female young adult (17-week-old) and old (19-month-old) mice, we investigated the effect of age in vivo on bones' adaptive response to loading and in vitro in primary cultures of osteoblast-like cells derived from bone cortices. Right tibias were axially loaded on alternate days for 2 weeks. Left tibias were non-loaded controls. In a separate group, the number of sclerostin-positive osteocytes and the number of periosteal osteoblasts were analyzed 24 hours after a single loading episode. The responses to strain of the primary osteoblast-like cells derived from these mice were assessed by EGR2 expression, change in cell number and Ki67 immunofluorescence. In young male and female mice, loading increased trabecular thickness and the number of trabecular connections. Increase in the number of trabecular connections was impaired with age but trabecular thickness was not. In old mice, the loading-related increase in periosteal apposition of the cortex was less than in young ones. Age was associated with a lesser loading-related increase in osteoblast number on the periosteal surface but had no effect on loading-related reduction in the number of sclerostin-positive osteocytes. In vitro, strain-related proliferation of osteoblast-like cells was lower in cells from old than young mice. Cells from aged female mice demonstrated normal entry into the cell cycle but subsequently arrested in G_2_ phase, reducing strain-related increases in cell number. Thus, in both male and female mice, loading-related adaptive responses are impaired with age. This impairment is different in females and males. The deficit appears to occur in osteoblasts' proliferative responses to strain rather than earlier strain-related responses in the osteocytes. © 2014 The Authors. *Journal of Bone and Mineral Research* published by Wiley Periodicals, Inc. on behalf of the American Society for Bone and Mineral Research.

## Introduction

Bones ensure that they are sufficiently robust to withstand the habitual levels of mechanical loading to which the skeleton is subjected without accumulating excessive microdamage, or sustaining frank fracture, by adapting their mass and architecture in response to their strain environment.^1^ This process is commonly called the mechanostat. We have hypothesized that the inadequate bone mass and increase in fragility fractures characteristic of the aged human skeleton represent an age-related impairment of the mechanostat.^2^

Interventional exercise studies in humans suggest that bones' periosteal modeling response to exercise declines with age in both sexes,^3^ although elderly men appear more responsive than women.^4^,^5^ In vivo studies in female rodents also suggest impairment of the osteogenic response to a defined strain-related stimulus.^6^,^7^ Perhaps controversially, this finding is not reproduced in aged male mice.^8^ Thus, in both human and the limited rodent studies, there appears to be evidence for sex-related differences in the effects of age on the mechanostat.

It is widely thought that osteocytes are one of the major cell types responsible for mechanosensation and that one of the ways in which they exert this strain-related influence is through their production of the Wnt antagonist sclerostin.^9^,^10^ Applying artificial loading to ulnae of transgenic mice overexpressing the human isoform of sclerostin, which is not downregulated after loading, abrogates the loading-related increase in mineral apposition rate.^11^ In humans, serum concentrations of sclerostin increase with age,^12^ suggesting a potential mechanism for the deleterious effects of age on bone's response to mechanical loading.

A recent in vitro study demonstrated that increasing concentrations of exogenous sclerostin can prevent the strain-related increase in proliferation of osteoblasts.^13^ Because these are the cells responsible for bone formation, their proliferative capability is another key mechanism required for bones' response to loading in vivo.^14^,^15^ Therefore, an age-related increase in sclerostin expression in osteocytes, that is not downregulated by strain, could prevent strain-related osteoblast proliferation and thus impair bones' osteogenic response to loading.

Although such a sclerostin-related mechanism could contribute to age-related impairment of bones' response to loading, it is unlikely to account for it totally because there are likely to be a number of additional age-related changes in both osteocytes and osteoblasts. For instance, osteoblast-like cells cultured in vitro from femoral explants of aged patients do not increase their proliferation after strain, unlike those from young controls.^16^ One of the earliest contributors to bone cells' responses to loading, likely to be involved in proliferation, is upregulation of the transcription factor early growth response factor (EGR)2.^17^ We have previously shown by microarray in loaded bones that EGR2 is a component of more strain-responsive pathways than any other transcription factor.^18^ In this respect, a change in EGR2 expression provides a useful indicator of any age-related impairment in bone cells' responses to strain.

In the study reported here, we determine the effect of age on the adaptive response to mechanical loading in the tibia of male and female mice. We characterize normal age-related change in bone structure and confirm that aspects of the adaptive bone response to loading are reduced with age, as is recruitment of osteoblasts to the periosteal surface, but we also show that loading-related osteocyte regulation of sclerostin is unaffected by age. In vitro age-related impairment of osteoblasts' strain-related response is demonstrable in cells from females. This may be owing to an increased incidence of arrest in the G_2_ phase of proliferation. Cells from males also show reduced strain-related replication, but in this case it cannot be ascribed to arrest in G_2_ but rather to impaired entry into the cell cycle.

## Materials and Methods

### Animals

Sixteen-week-old and 19-month-old male and female C57BL/6 mice (*n* = 53) were obtained from Charles River Inc. (Margate, UK). All mice were allowed free access to water and a maintenance diet containing 0.75% calcium (EURodent Diet 22%; PMI Nutrition International, LLC, Brentwood, MO, USA) in a 12-hour light/dark cycle, with room temperature at 21 ± 2°C. All cages contained wood shavings, bedding, and a cardboard tube for environmental enrichment. Female mice were housed in groups of up to 5 animals, whereas males were housed individually to prevent fighting and its effect of bone mass.^19^ All procedures complied with the UK Animals (Scientific Procedures) Act 1986 and were reviewed and approved by the University of Bristol ethics committee (Bristol, UK).

### Ex vivo strain measurements

To apply similar magnitudes of peak strain in young and aged male and female mice, we first established the load:strain relationship ex vivo in a subsample of male and female young and aged mice (*n* = 5). In each mouse, a single element strain gage (EA-06-015DJ-120, Vishay Measurement Group, Wendell, NC, USA) was bonded longitudinally to the medial aspect of the right tibia at 37% of its length from the proximal end. This is the site where we have previously observed the greatest osteogenic response to axial loading.^20^–^22^ Although strain was only measured at a single site, a recent publication reported that aging does not substantially affect the position of the strain neutral axis (and therefore strain distribution) despite a change in bone morphology.^23^ Strains were measured across a range of peak loads between 5 and 17 N, applied using the same electromagnetic loading machine used for in vivo loading (ElectroForce 3100; Bose Co., Eden Prairie, MN, USA). Linear regression analysis allowed calculation of the loads required to apply 500, 1000, 1500, 1750, 2000, 2250, and 2500 με at the start of the study (Supplemental Table S1).

### In vivo external mechanical loading

Right tibias were subjected to external mechanical loading under isoflurane-induced anesthesia on alternate days for 2 weeks to investigate the effect of loading on structural bone adaptation (*n* = 42, 6 in each of 7 strain groups) or once only to determine the effect of loading on osteocyte sclerostin and periosteal osteoblast number 24 hours later (*n* = 6). Left limbs were used as internal controls as previously validated.^21^,^24^ The protocol for noninvasively loading the mouse tibia has been reported previously.^21^,^25^ In brief, the flexed knee and ankle joints are positioned in concave cups; the upper cup, containing the knee, is attached to an actuator arm of a loading device and the lower cup to a dynamic load cell. The tibia is held in place by a 0.5-N continuous static preload. Forty cycles of dynamic load are superimposed with 10-second rest intervals between each cycle. The protocol for one cycle consists of loading to the target peak load, hold for 0.05 seconds at the peak load, and unloading back to the 0.5-N preload. From the strain gage data (see “ex vivo strain measurements”), different peak loads for young and aged males and females were calculated as shown in Supplemental Table S1. Strain rate at this site was normalized to a maximum of 30,000 μεs^−1^, also shown in Supplemental Table S1.

### High-resolution μCT analysis

Because the mouse bone is small and the present axial loading-related osteogenesis is site-specific,^9^,^21^ high-resolution μCT was used to quantify three-dimensional bone architecture at precisely comparable sites of the loaded and contralateral control tibias. After euthanization, lower legs were stored in 70% ethanol and whole tibias imaged using the SkyScan 1172 (SkyScan, Kontich, Belgium) with a voxel size of 4.8 µm (110 μm^3^). The scanning, reconstruction, and method of analysis has been previously reported.^19^,^26^ We evaluated the effect of age and sex on both tibias and changes ([right – left] / left)*100 resulting from loading in bone volume fraction (BV/TV), trabecular thickness (Tb.Th), trabecular number (Tb.N), and trabecular pattern factor (Tb.Pf) in the trabecular region (0.25 to 0.75 mm distal to the proximal physis) and cortical bone area (Ct.Ar), total cross-sectional area inside the periosteal envelope (Tt.Ar), medullary area (Ma.Ar), cortical area fraction (Ct.Ar/Tt.Ar), and cortical thickness (Ct.Th) at the cortical site (37% from the proximal end), according to ASBMR guidelines.^27^ Trabecular pattern factor was used to measure trabecular connectivity as previously validated^28^ with a more negative value indicating an increased connectivity.

### Histology

Twenty-four hours after a single episode of loading with peak strain magnitude of 2500 με (equivalent to approximately 6000 με on the posterior-lateral surface), mice were killed and bilateral tibias fixed in 4% paraformaldehyde (PFA) for 48 hours at 4°C and then decalcified for 21 days in 14% EDTA with continuous agitation. The solution was changed three times per week and adequate decalcification confirmed by imaging using micro-computed tomography (μCT) and comparing the bone density with surrounding muscle. Bones were then processed for histology and wax embedded and sectioned transversely with 8-μm thickness. Sections corresponding to the 37% site of the tibia (measured from the proximal end and where bone formation after mechanical loading is maximal^20^–^22^) were stained using a standard hematoxylin and eosin (H&E) staining protocol or using the sclerostin or periostin immunostaining protocol. This was as previously described.^9^,^29^ Periostin is preferentially expressed by osteoblasts, so it was used to validate that cells located in the periosteum that were counted on the H&E-stained sections were osteoblastic in nature (Supplemental Fig. S1).

Slides were imaged in the posterior-lateral region where strains have previously been shown to be maximal by finite element analysis^9^ using the 40× objective lens and cells were counted manually, using ImageJ to record numbers of positive and negative cells. One field of size 220 × 166 μm was counted on a minimum of four sections for each mouse by two independent observers. This contained on average 54 lacunae per section analyzed.

### In vitro experiments

Cortical long bone-derived osteoblast-like primary cells were cultured from pooled bone chips obtained from both forelimbs of 2 to 3 of the same mice used for the loading studies using a well-established technique.^13^,^17^,^30^–^32^ This approach was taken to reduce the numbers of aged animals required for these experiments. Osteoblasts were cultured for 2 to 4 weeks until reaching 90% confluency. All cells were used at passage 1. Alkaline phosphatase activity and the ability to form mineralized nodules were used to confirm the osteoblastic nature of the cultured cells (Supplemental Fig. S2) as previously described.^13^ Cells were seeded onto custom-designed plastic strips for proliferation and qRT-PCR studies as previously described.^13^,^17^,^30^–^32^ For proliferation studies (cell number and Ki67 immunofluorescence), cells were seeded at 10,000 cells/cm^2^. For qRT-PCR, cells were seeded at 25,000 cells/cm^2^. Mechanical strain was applied in a ramped square wave by four-point bending of the substrate on which the cells were grown. Cells were subjected to 600 cycles of four-point bending engendering a peak strain magnitude of 3400 με, a strain rate on and off of 23,000 μεs^−1^, and a dwell time in load of 0.7 seconds and between cycles of 0.7 seconds, giving a frequency of 0.6 Hz. Cells were fixed for analysis by DAPI staining to count cell nuclei number (48 hours after strain), Ki67 immunofluorescent staining to count the proportion of actively proliferating cells (24 hours after strain), and lysed in RNA lysis buffer to analyze EGR2 and beta-2 microglobulin (B2MG) by qRT-PCR (1 hour after strain). All methods have been previously described.^13^,^17^,^30^–^32^ The number of technical repeats (*n*) and independent biological repeats are explicit in the relevant figure legends.

### Statistical analysis

To analyze the effect of aging on parameters of bone mass and architecture, a one-way ANOVA was used with Bonferonni post hoc correction. To analyze the effect of aging on load-engendered strain, linear regression analysis was used to compare lines. Two-stage linear regression was used to determine the effect of aging on the loading response of bone and the inflection point (corresponding to the minimum effective strain [MES]) and slope compared post hoc using *t* tests if the overall effect of age was significant. Where it was not possible to fit a two-stage regression model, a linear regression was performed and the gradient of the line compared with zero. Paired *t* tests were used to evaluate the effect of loading on paired left control and right loaded samples. Unpaired *t* tests were used to assess the effect of age within each sex. All statistics were performed using GraphPad Prism version 6.0 for Mac (GraphPad Software, La Jolla, CA, USA).

## Results

### Age is associated with less robust cortical and trabecular bone architecture in mice

The effect of age on cortical and trabecular bone mass and architecture in tibias of male and female 17-week-old young adult and 19-month-old aged C57Bl/6 mice was established using μCT. As expected from previous studies in other mouse long bones,^33^–^35^ trabecular bone volume fraction was significantly lower in old mice of both sexes in the proximal tibia (male –43%, female –77%, *p* < 0.001). This was because of a reduction in trabecular number (male –51%, female –78%, *p* < 0.001), which was uncompensated for (in terms of BV/TV) by a small but significant increase in the thickness of remaining trabeculae (male 17%, female 16%, *p* < 0.001). Age did not affect the trabecular pattern factor, a measure of connectivity, in male mice (3.1%, *p* = 0.38) but led to an age-related increase in female mice (23%, *p* < 0.001), indicating a sex-specific reduction in connectivity. These data are summarized in Table 1.

**Table 1:** Aging Has Detrimental Effects on Trabecular and Cortical Bone Mass and Architecture in the Murine Tibia

Sex	Male		Female	
Age	Young adult	Aged	Young adult	Aged
Body weight (g)	28 ± 0.3	**34 ± 0.4**	22 ± 0.2	**27 ± 0.3**
Tibial length (mm)	18 ± 0.04	**18 ± 0.04**	18 ± 0.04	**18 ± 0.04**
Trabecular bone				
BV/TV (%)	9.0 ± 0.2	**5.1 ± 0.2**	6.5 ± 0.2	**1.5 ± 0.5**
Tb.Th (mm)	0.037 ± 0.0004	**0.043 ± 0.0006**	0.046 ± 0.0006	**0.054 ± 0.002**
Tb.Pf (mm^−1^)	31 ± 0.6	30 ± 0.9	34 ± 0.6	**42 ± 2**
Tb.N (mm^−1^)	2.4 ± 0.06	**1.2 ± 0.06**	1.4 ± 0.04	**0.31 ± 0.1**
Cortical bone				
Ct.Ar (mm^2^)	0.76 ± 0.01	**0.62 ± 0.007**	0.67 ± 0.006	**0.55 ± 0.007**
Tt.Ar (mm^2^)	1.4 ± 0.02	1.4 ± 0.02	1.2 ± 0.01	1.2 ± 0.01
Ma.Ar (mm^2^)	0.65 ± 0.01	**0.77 ± 0.02**	0.50 ± 0.005	**0.59 ± 0.01**
Ct.Th (mm)	0.13 ± 0.001	**0.10 ± 0.002**	0.14 ± 0.001	**0.11 ± 0.02**

Parameters of bone mass and architecture were measured in trabecular bone of the proximal tibia (0.25 to 0.75 mm distal to the proximal physis) and cortical bone (37% site measured from the proximal end) using CT. Data shown as mean ± SEM, *n* = 42. Parameters in bold were significantly different attributable to aging; *p* < 0.001.

In cortical bone, both old male and female mice had a lower cortical bone area (male –19%, female –18%, *p* < 0.001) than in young mice. This was the result of an increase in medullary area (male 20%, female 20%, *p* < 0.001) with no overall change in total (periosteally enclosed) tissue area (male –1.0%, *p* = 0.59; female –1.7%, *p* = 0.24). This suggests an age-related expansion of the medullary cavity with no overall change in periosteal perimeter. These changes resulted in a significant age-related decrease in overall cortical thickness (male –24%, female –19%, *p* < 0.001).

### Age is associated with reduced bone stiffness

**Fig 1:**
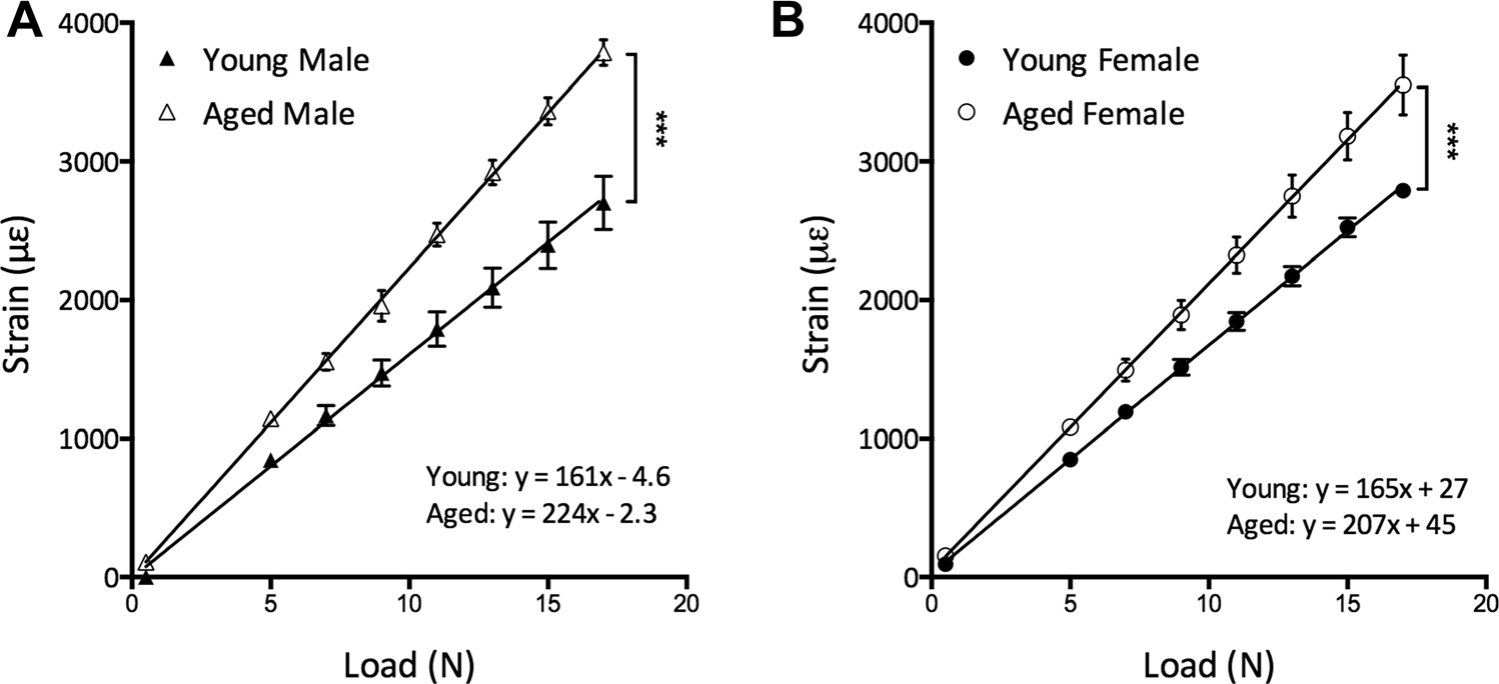
Tibial stiffness is reduced with aging in mice. Loading-engendered strains were measured on the medial surface of the tibia at the 37% site (measured from the proximal end) in males (*A*) and females (*B*) of both ages. Data represent mean ± SEM, *n* = 5. ***p < 0.001 by linear regression analysis.

In both male and female mice, the gradient of the load:strain regression line was significantly steeper in aged animals, indicating a reduction in stiffness that would result in higher strains being engendered from the same loads (*p* < 0.001, Fig. 1*A*, *B*). These load:strain data were used to calculate the loads required to engender similar strains in the different groups of mouse (Supplemental Table S1).

### Age does not affect loading-related increase in trabecular thickness but is associated with lack of increase in trabecular connectivity

**Fig 2:**
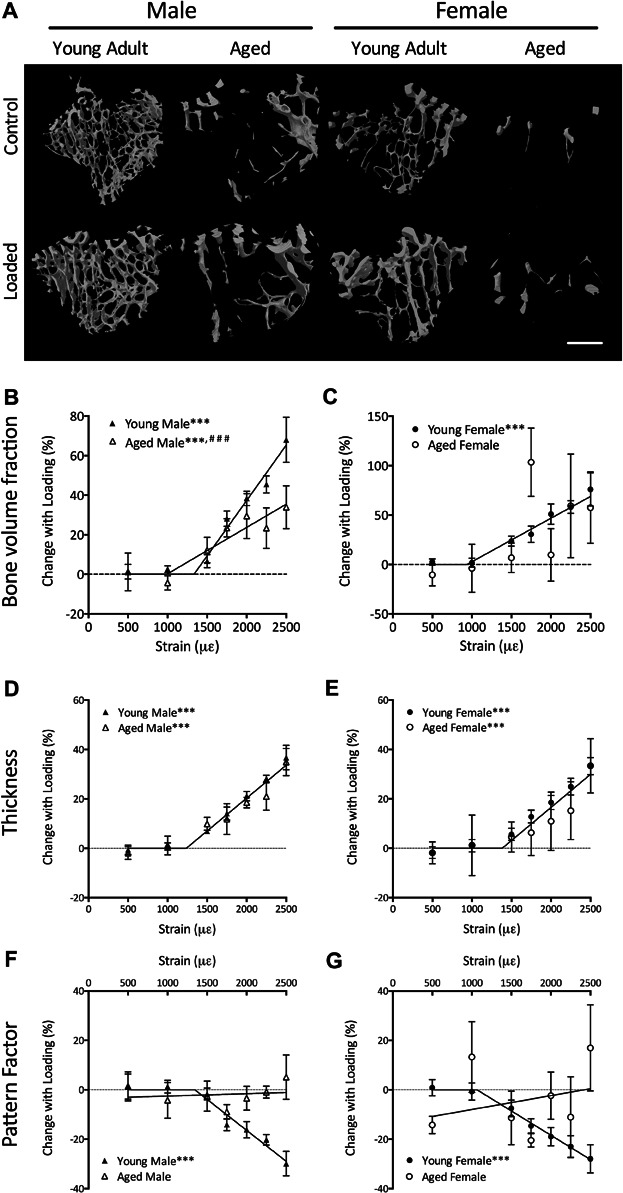
The loading-related increase in trabecular thickness is unaffected by aging, but the increase in trabecular connectivity is abrogated in male and female mice. (*A*) Representative 3D reconstructions showing the effect of loading on the region of trabecular bone analyzed by μCT. Scale bar = 500 μm. Percentage change ([right – left] / left) * 100 in trabecular bone volume fraction (*B*, *C*), trabecular thickness (*D*, *E*), and pattern factor (*F*, *G*) were compared in young and aged mice. Data represent mean ± SEM, *n* = 6 for each strain magnitude. It was not possible to fit a two-stage linear regression for trabecular pattern factor in aged mice, and linear regression was not significantly different from 0. ***p < 0.001 for a gradient of the load-response regression line being different from 0. ^###^*p* < 0.001 overall difference between young and aged lines by regression analysis.

Trabecular thickness increased with loading in a peak strain magnitude-dependent manner in both young and aged male and female mice once a strain threshold (the MES) had been exceeded (Fig. 2*D*, *E*). No difference in the trabecular thickness response was detected between young and aged mice of the same sex (male *p* = 0.38, female *p* = 0.45).

Because of the marked age-related reduction in trabecular number (Supplemental Fig. S3), and therefore bone volume fraction (Fig. 2*B*, *C*), the variation between individuals meant that interpretation of the response of these parameters to loading was problematic. However, previous studies have demonstrated the predominant effect of loading on trabecular bone to be an increase in trabecular thickness^20^,^21^ and, because this parameter is normalized to individual trabeculae, the age-related variation was reduced.

The trabecular pattern factor decreased strain-dependently with mechanical loading in young male and female mice once the MES was exceeded, indicating the formation of new trabecular connections after loading (Fig. 2*F*, *G*). This response was absent in aged mice because the linear regression lines between applied strain magnitude and loading-related change in pattern factor were not significantly different from zero (male *p *= 0.77, female *p *= 0.74, Fig. 2*F*, *G*).

### Age is associated with lower loading-related cortical bone formation in both male and female mice

**Fig 3:**
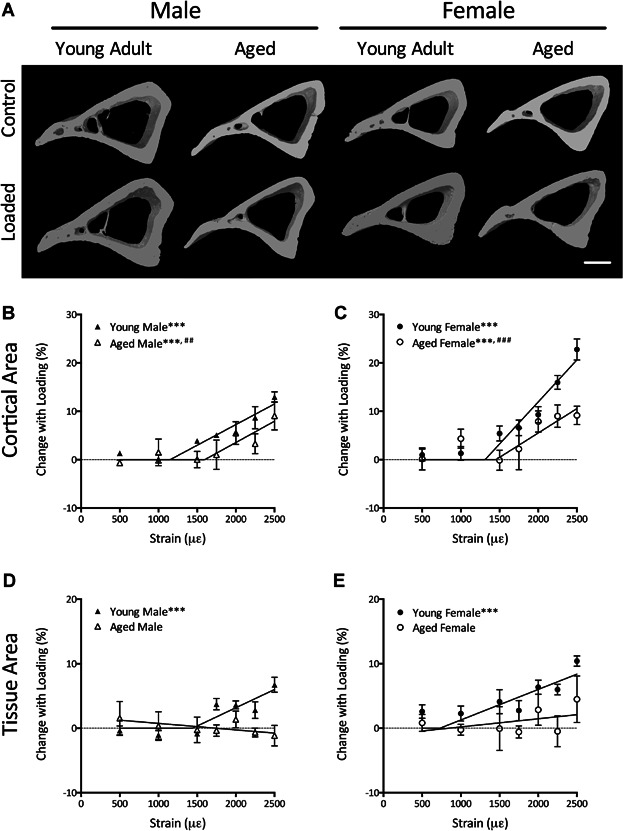
Aging impairs cortical bone formation because of prevention of periosteal apposition. (*A*) Representative 3D reconstructions showing the effect of loading on the region of cortical bone analyzed by μCT. Scale bar = 500 μm. Percentage change ([right – left] / left) * 100 in cortical bone area (*B*, *C*) and total tissue area inside the periosteal envelope (*D*, *E*) were compared in young and aged mice. Data represent mean ± SEM, *n* = 6 for each strain magnitude. It was not possible to fit a two-stage linear regression for tissue area in aged mice, and linear regression was not significantly different from 0. ***p < 0.001 for a gradient of the load-response regression line being different from 0. ^##^*p* < 0.01, ^###^*p* < 0.001 overall difference between young and aged lines by regression analysis.

In male mice of both ages, cortical bone area increased with loading. Overall, there was a significant reduction in the response to loading in aged male mice compared with young adults (*p* < 0.01, Fig. 3*B*), but the slope of the strain-response curve did not change with age (*p* = 0.98). The MES was 470 με higher in aged compared with young males, although this difference was not statistically significant (*p* = 0.14).

In females, there was also a significant increase in cortical area in both ages of mouse, but again, the increase was significantly lower in aged animals (*p* < 0.001). There was no age-related difference in the threshold above which cortical bone area increased (*p* = 0.89). However, once the threshold was exceeded, the gradient of the slope was significantly lower in aged female mice compared with young females (*p* < 0.05, Fig. 3*C*), indicating a lower osteogenic response.

As previously reported,^20^,^21^ loading produced a linear dose-dependent increase in the total tissue area inside the periosteal envelope, indicating periosteal bone formation in young male and female mice (Fig. 3*D*, *E*). However, in aged mice, no significant difference in tissue area was observed between left control and right loaded limbs at any strain magnitude, and the linear regression line was not significantly different from 0 (male *p *= 0.30, female *p* = 0.36). This indicates an age-related reduction in strain-related periosteal bone formation after mechanical loading, which is consistent with that found in previous studies.^36^

There was no significant effect of loading on the area of the medullary cavity in any age or sex of mouse. However, in aged mice, there was a decrease in medullary area at higher magnitudes of mechanical strain. Cortical thickness increased with loading, but no significant difference was detected associated with age. These data are shown in Supplemental Fig. S4.

### Age has no effect on loading-related change in the proportion of sclerostin-positive osteocytes but is associated with reduced increase in periosteal osteoblast number

**Fig 4:**
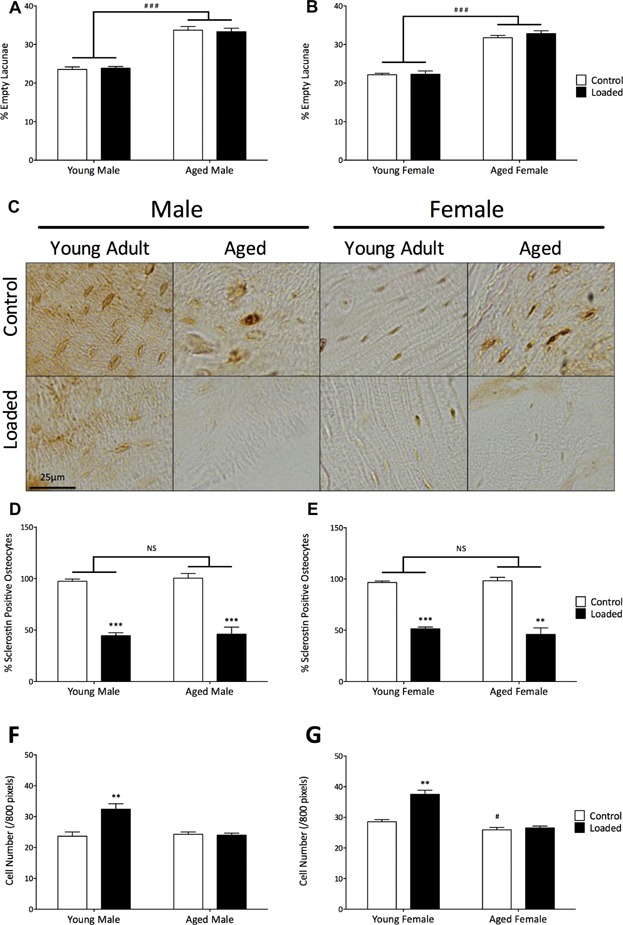
Loading-related osteocytic sclerostin downregulation was not affected by aging, but the increase in periosteal cell number was abrogated. (*A*, *B*) The number of empty osteocyte lacunae were counted in 6 H&E-stained sections in 5 mice. (*C*) Representative sclerostin immunohistostaining and (*D*, *E*) quantification in 6 sections from 5 mice per group 24 hours after loading. (*F*, *G*) Periosteal cell numbers were counted in H&E-stained sections. **p < 0.01, ***p < 0.001 by paired Student's *t* test to determine the effect of loading; ^#^*p *< 0.05 versus young female controls (by unpaired Student's *t* test).

We next sought to examine whether loading-related sclerostin regulation in osteocytes was affected by age because this process appears to be required for bone to adapt to its mechanical environment.^11^ Because previous studies have shown that osteocyte apoptosis increases with age,^37^ we needed to first establish the proportion of lacunae that did not contain an observable osteocyte cell body so that these lacunae were not mistakenly counted as negatively stained osteocytes. As expected, the percentage of empty lacunae increased with age in both sexes (male 33% increase, female 31% increase, *p* < 0.001, Fig. 4*A*, *B*). No change in the percentage of empty lacunae was observed 24 hours after a single period of loading.

Loading caused a significant decrease in the number of sclerostin-positive osteocytes in all groups of mice (young male –54%, aged male –54%, young female –47%, aged female –53%, *p* < 0.001, Fig. 4*C*) as previously reported in young mice.^9^,^10^ After correcting for the increased number of empty lacunae in aged mice, no significant difference in loading-related sclerostin downregulation was detected attributable to age in either sex (male *p* = 0.92, female *p* = 0.49, Fig. 4*D*, *E*).

We then investigated whether age affected the subsequent loading-related increase in periosteal cell number. A small but significant age-related decrease in the number of periosteal cells in the left non-loaded limbs was observed in females but not males (Fig. 4*F*, *G*). In young adult male and female mice, we observed a significant increase in periosteal cell number 24 hours after loading (male 29%, *p* < 0.01; female 32%, *p* < 0.001, Fig. 4*F*, *G*, and Supplemental Fig. S1), as previously described in other models, albeit at different time points.^14^,^15^,^38^ Interestingly, there was no further increase by 3 days after loading (data not shown). However, in aged mice, there was no significant loading-related increase in cell number (male –2.8%, *p* = 0.65; female 4.4%, *p* = 0.18, Fig. 4*F*, *G*). Immunohistostaining confirmed that the cells within the periosteum counted for this analysis were periostin-positive, indicating they were osteoblastic in nature (Supplemental Fig. S1).

### Age reduces strain-related proliferation in osteoblast-like cells from both male and female mice in vitro

**Fig 5:**
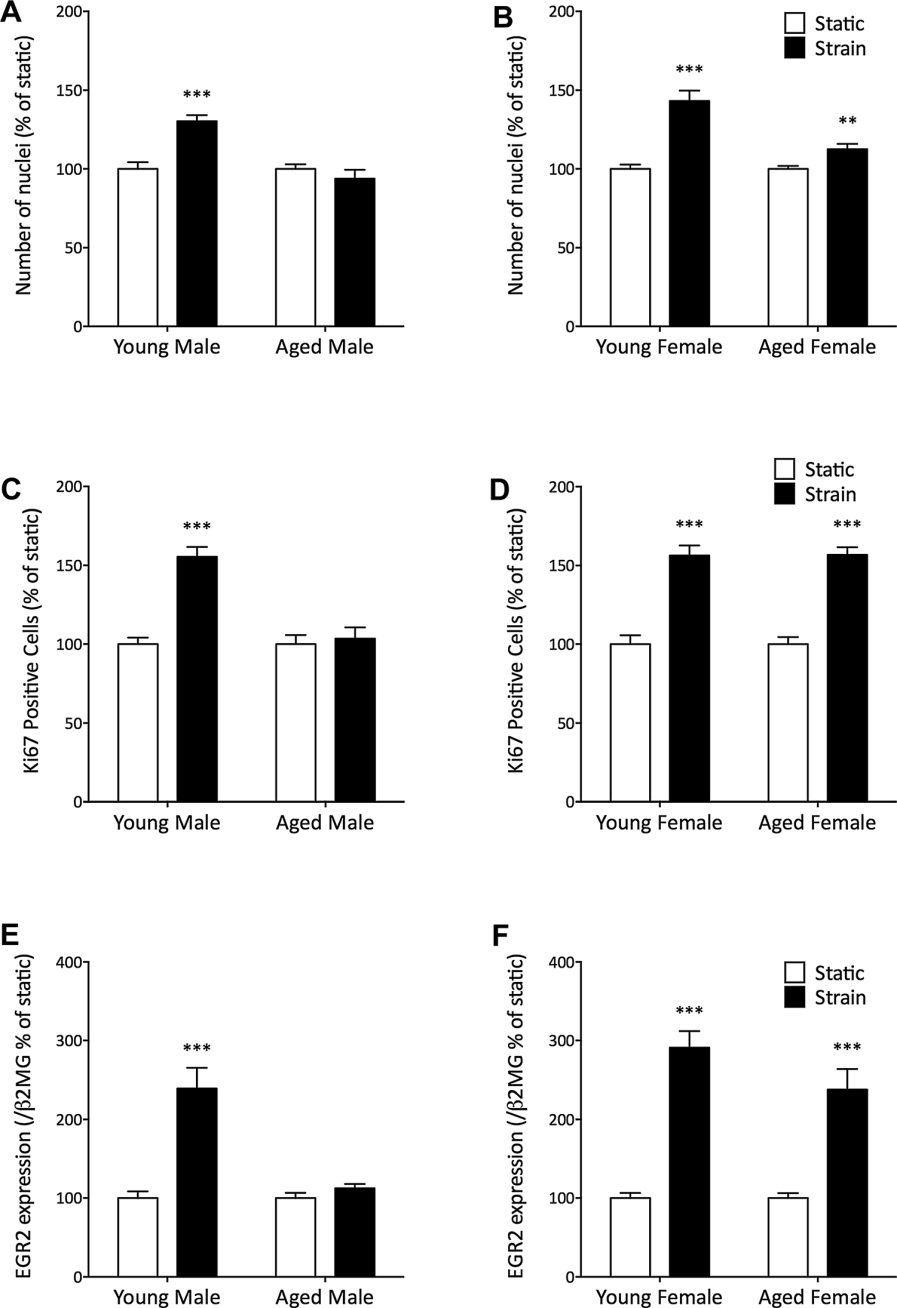
Aging reduces the strain-related increase in proliferation of osteoblast-like cells. (*A*, *B*) Cell number was counted 48 hours after strain by analyzing nuclear DAPI staining, *n* = 6 per group with 4 repeats. (*C*, *D*) Proportion of cells stained positive for Ki67 were analyzed 24 hours after strain using immunofluorescence, *n* = 4 with 3 repeats. (*E*, *F*) EGR2 expression was quantified by qRT-PCR 1 hour after strain, *n* = 6 with 3 repeats. Bars represent mean and SEM. ***p < 0.001 by paired Student's *t* test to determine the effect of strain.

Because age abrogated the loading-related increase in periosteal bone area and periosteal osteoblast number, we next assessed the cell autonomous effects of age on the proliferative response of osteoblast-like cells to mechanical strain in vitro. The osteoblastic nature of these cells was confirmed by their expression of alkaline phosphatase (ALP) and ability to form mineralized nodules (Supplemental Fig. S2). A significant increase in cell number was observed 48 hours after application of strain in osteoblast-like cells derived from both young males (30% increase, *p* < 0.001, Fig. 5*A*) and females (43%, *p* < 0.001, Fig. 5*B*). There was no significant strain-related change in the number of cells from aged males (–6.2%, *p* = 0.33, Fig. 5*A*), but there was a smaller (13%, *p* < 0.01), and significantly diminished, increase in cells from aged females when compared with those from young females (*p* < 0.001, Fig. 5*B*).

To analyze this difference more closely, we used in situ cell cycle analysis, by immunofluorescent staining for the proliferation marker Ki67.^13^,^39^,^40^ This allowed us to determine the percentage of actively proliferating cells. Consistent with the strain-related change in cell number, there was a significant increase in the percentage of Ki67-positive cells from young male (56%, *p* < 0.001, Fig. 5*C*) and female (56%, *p* < 0.001, Fig. 5*D*) mice but no such strain-related increase in the percentage of Ki67-positive cells from aged male mice (3.4%, *p* = 0.71, Fig. 5*C*). Unexpectedly, the increase in the percentage of aged female Ki67-positive cells after strain (57%, *p* < 0.001) was not statistically different from that seen in young female osteoblasts (*p* = 0.96, Fig. 5*D*). This contrasts with the age-related reduction in the strain-related increase in cell number (Fig. 5*B*).

We then investigated the effect of strain on EGR2. Consistent with the previous data, EGR2 expression was increased with strain in cells from young male (140%, *p* < 0.001, Fig. 5*E*) and female (190%, *p* < 0.001, Fig. 5*F*) mice. Strain-related EGR2 upregulation in cells from aged female mice (140%, *p* < 0.001) was not significantly different from that observed in young females (*p* = 0.13, Fig. 5*F*). In contrast, there was no strain-related increase in expression in osteoblasts from aged male mice (1.8%, *p* = 0.86, Fig. 5*E*).

### Sex-specific delays in cell-cycle recruitment and progression are observed after strain in osteoblast-like cells from aged mice

**Fig 6:**
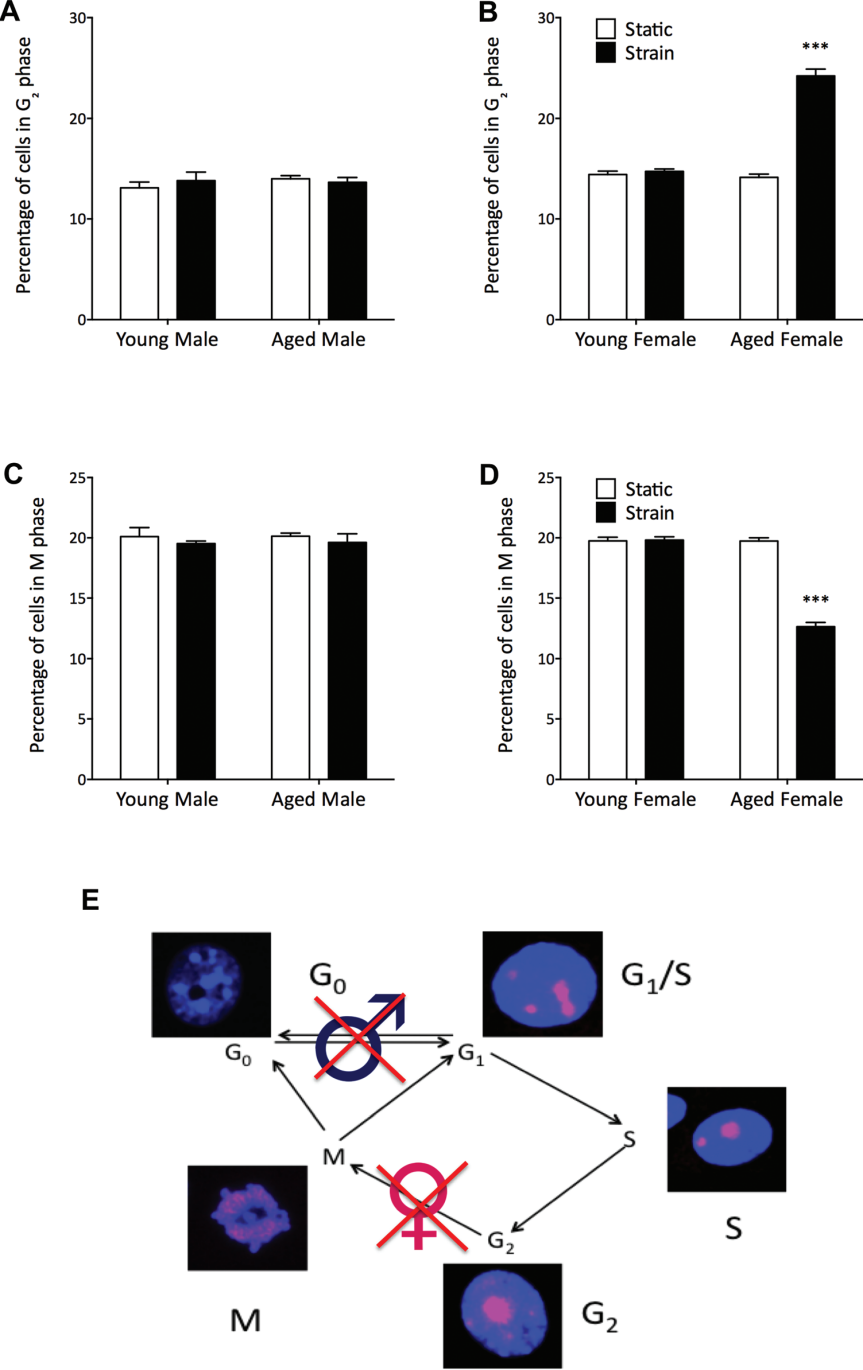
Aging causes a strain-related G_2_-phase cell-cycle arrest in osteoblast-like cells from female, but not male, mice. Proportion of Ki67-positive cells in G_2_ phase (*A*, *B*) and M phase (*C*, *D*) of the cell cycle were calculated by analysis of Ki67 nuclear patterning (*E*) using immunofluorescence, *n* = 4 per group with 2 repeats in males and 3 repeats in females. Bars represent mean and SEM. ***p < 0.001 by paired Student's *t* test to determine the effect of strain.

To further analyze changes in proliferation after strain, the pattern of Ki67 nuclear localization was examined to determine the stage of the cell cycle that each proliferating cell was in.^13^,^39^,^40^ In male osteoblast-like cells, there was no difference in the number of cells in each stage of the cell cycle between static and strained, young or aged osteoblasts (Fig. 6*A*, *C*, and Supplemental Fig. S5). Given the lack of increase in Ki67 positivity after strain in cells from aged male mice, this indicates a failure of cells in aged animals to be recruited from a quiescent state into the cell cycle (Fig. 6*E*).

In contrast, in cells from aged female mice, we did find a strain-related increase in the proportion of Ki67-positive cells in G_2_ phase of the cell cycle (71%, *p* < 0.001, Fig. 6*B*), with a corresponding decreased proportion of cells in M phase after strain (–36%, *p* < 0.001, Fig. 6*D*) when compared with young female osteoblasts. This is consistent with an arrest of cells in G_2_ phase of the cell cycle after strain, preventing their progression into M phase. This could explain why cells from aged female mice show an attenuated increase in cell number relative to those from young female mice despite being similarly recruited to the cell cycle.

## Discussion

In this study, we report that aged mice have a less robust trabecular and cortical bone mass and architecture than young mice. Our findings suggest that this could, at least in part, be the result of impaired strain-related osteoblast proliferation associated with sex-specific perturbations in cell-cycle entry and progression after stimulation by mechanical strain.

Our study of normal bone architecture in young and old mice indicated that, as in humans, age itself is associated with a significant decline in skeletal robustness in both males and females. This is the result of a dramatic reduction in trabecular number accompanied by only a mild increase in trabecular thickness. This is consistent with data from the femora of mice during aging^33^–^35^ and is similar to that reported in humans.^41^ Additionally, in our study, there was an age-related increase in trabecular pattern factor in female, but not male, mice, despite a similar loss of trabecular bone volume fraction in both sexes. This also mirrors findings in iliac crest biopsies in humans.^28^ Together these data suggest that mice undergo a similar deterioration of skeletal structure to humans, which reinforces the suitability of using the mouse as a model for human skeletal aging.

As expected, loading that engendered strains lower than those experienced during customary activity produced no adaptive increases in bone mass. However, once this threshold is exceeded, the strain dose:bone mass response is linear, as we have reported previously.^20^ After 2 weeks of mechanical loading, trabecular thickness increased in both young and old mice with no difference between them. To our knowledge, the only other study that examined the effect of loading on trabecular bone in aged mice demonstrated that loading was associated with a significant loss of trabecular bone in young males but prevented the age-related loss of trabecular bone in aged males.^8^ One possible explanation was that this study used a low strain rate, which had previously been observed to cause a loss of trabecular bone.^25^ In contrast, the physiological strain rate used in our current study has previously been documented to be associated with increased trabecular bone formation.^19^,^21^,^22^ Although attempts were made to strain-match mice with differing bone phenotypes in the cortical region, we were not able to quantify strains in the trabecular region. Such quantification would involve introducing so many assumptions that we consider it unsafe. In the absence of specific data on trabecular strains, it is therefore impossible with any certainty to state that the strain-related stimulus is equivalent in the different groups. This is an inherent deficiency in the methodologies currently available. Interestingly, there was no significant loading-related increase in trabecular number in aged mice, nor a significant decrease in trabecular pattern factor, suggesting that formation of new trabecular connections in aged mice is significantly reduced. Of course, the strain-related response of individual trabeculae is problematic. If increase in the number of trabeculae is reduced with age and the increase in trabecular thickness is unaffected, presumably the overall strain-related response per trabecula must be reduced.

In cortical bone, a diminished response to loading was observed in both male and female aged mice. This appeared to be related to sex; in male mice the difference (to the extent that it exists not being statistically significant) could reflect an age-related increase in the threshold of strain sufficient to engender an osteogenic response. In males, there was no age-related change in the slope of the response, whereas in females, there was a significant age-related decrease in the slope of the response but no age-related change in the threshold at which an osteogenic response occurred. This suggests that the strain threshold for stimulating new bone formation tends to increase with age in males, whereas in females, the threshold is no different but the consequent response is impaired. These data on the threshold at which an osteogenic response is engendered could be associated with different activity levels.^19^ However, if so, this would imply that male, but not female, mice experience an age-related increase in activity. This would be counterintuitive.

That in the cortical regions of the bone in both aged males and females there should be no loading-related increase in the tissue area enclosed within the periosteum suggests that this response is impaired with age. A limitation of our study was the absence of dynamic measures of bone formation using histomorphometry or in vivo μCT scanning and the reliance on side-to-side comparisons. Although these methods would provide additional information, we do not think that these would alter the implications of the data presented here. Furthermore, previous studies have already demonstrated an age-related prevention of periosteal with enhanced endosteal bone formation after mechanical loading in aged mice using dynamic histomorphometry,^36^ and our results are consistent with these data. A small amount of periosteal woven bone formation was observed in some young mice in the highest strain group only, which is to be expected and reflects a rapid response to a potent stimulus.

To determine at which stage of the adaptive response to mechanical loading this impairment occurred, we examined age and loading-related changes in a number of previously reported responses of bone and bone cells to mechanical loading. In both male and female mice, osteocytic downregulation of sclerostin was unaffected by age. Although downregulation of sclerostin is only one cellular process involved in bone's osteogenic response to mechanical loading, it appears to be necessary for subsequent adaptive new bone formation.^11^ However, because the process by which osteocyte sclerostin actually influences Wnt signaling in osteoblasts remains unclear, we cannot exclude the possibility that, with fewer viable osteocytes present in aged bone, the change in sclerostin concentration engendered by viable osteocytes may not reflect the sclerostin concentration experienced by osteoblasts at the bone surface, where new bone formation takes place.

Nevertheless, the absence of any age-related change in sclerostin with strain suggests that it is the loading-related response of osteoblasts, rather than that of osteocytes, that is detrimentally affected by age. Previous studies document a depletion of the pool of osteoprogenitor cells with age attributable to activation of adipogenesis at the expense of osteoblastogenesis.^34^,^42^–^44^ Therefore, in aged bone, it may not be possible to increase rates of osteoblastogenesis after loading despite osteocytic sclerostin downregulation. This also provides a potential cellular explanation for the lack of periosteal bone formation to mechanical loading observed in vivo because osteoblast proliferation is likely to be a key step in bone's adaptation to mechanical loading.^14^,^15^,^38^

To examine the role of strain on osteoblasts in more detail, we cultured these cells from mouse long bone chips and assessed their responses to mechanical strain applied in vitro in the absence of systemic endocrine and paracrine influences. Caution must, therefore, be made when extrapolating the findings of these studies to the in vivo context. In primary cells from aged male mice, strain had no effect on the early regulation of EGR2 expression, the number of actively proliferating cells, or the subsequent increase in cell number. This suggests that the increased threshold at which strain engenders an adaptive response in vivo in males is associated with a decreased responsiveness to strain rather than being related to the animals' habitual activity. It would be interesting to test this by increasing the magnitude of the mechanical strain stimulus in vitro, but this is not possible for technical reasons because the plastic slides we use for strain application start to crack if bending is increased further.

In cells from female mice, the early responses to mechanical strain including upregulation of EGR2 and the strain-related increase in the proportion of proliferating cells were also unaffected by age. However, the strain-related increase in cell number was reduced. Examination of cell-cycle stages of proliferating cells indicated that this was associated with an increase in the proportion of cells in G_2_ phase and a decrease in those in M phase. This suggests that although recruitment of osteoblastic cells to the cell cycle (and thus all the steps preceding that) is not affected by age, there is a degree of age-related arrest during progression through the cell cycle. Interestingly, recent studies have indicated that senescent cells can arrest in G_2_ phase of the cell cycle^45^,^46^ and that the proportion of senescent cells increases with age,^47^ providing a potential mechanism for this observation.

Taken together, these findings suggest that in male and female mice, there is a reduction in the strain-related increase in cell number after strain. However, the impediment to increased cell number differs between males and female. In males, a number of processes preceding division (represented by increased EGR2 expression) appear to be deficient and the cells do not appear to be recruited to the cell cycle (as indicated by Ki67). In females, these processes seem to be unaffected by age (as indicated by unchanged EGR2 expression) and cells enter the cell cycle (as indicated by Ki67) but replication is arrested in the G_2_ stage of the process.

In conclusion, we have demonstrated that in adulthood, increasing age is associated with reduction in cortical and trabecular bones' adaptive responses to artificial loading in both male and female mice. Although the quantitative strain:response relationship in vivo is unaffected by age in males, in females similar increases in strain in old mice stimulate less new bone formation than in young ones. Although there are fewer viable osteocytes in old than young bones, there appears to be no age-related change in osteocytes' response to strain, as indicated by changes in their sclerostin positivity. In vitro studies suggest that in both males and females, strain-related increase in osteoblast number is reduced with age. In males, this appears to be the result of reduced recruitment to the processes of division, whereas in females, it is attributable to arrest at the G_2_ stage of the process. This suggests that therapy to restore bones' adaptive responsiveness to load bearing in the old could usefully be directed toward enhancing the proliferative responsiveness of osteoblasts rather than the strain-related responsiveness of osteocytes.

## Disclosures

All authors state that they have no conflicts of interest.

## Supporting Information

Supplementary Figure 1 Loading increased the number of osteoblastic cells in the periosteum of young but not old mice. (A) Representative H&E sections from the posterior-lateral (high strain) region of the tibia of male mice 24 hours after artificial loading. Cells were counted in the periosteum which is indicated by the open brackets. (B) Periostin staining was used to confirm that the cells counted were osteoblastic as illustrated in a representative high power image. Scale bar = 25m.

Supplementary Figure 2 Cells cultured from aged mice are osteoblastic in nature as shown by their expression of alkaline phosphatase and their ability to form mineralized nodules. (A) ALP activity was measured between 7 and 21 days in osteoblastic cells cultured from the long bones of aged male and female mice. Results are corrected for protein content and expressed as a percentage of ALP activity at day 7. (B) After 21 days in culture in complete medium containing 50µM ascorbic acid and 10mM β-glycerol phosphate, cells from young and aged mice formed mineralized nodules as shown by Alizarin Red staining. Scale bar = 15mm.

Supplementary Figure 3 The effect of loading on trabecular bone in young and aged male and female mice. Percentage change [(right - left) / left] * 100 in trabecular number (A) and separation (B) were compared in young and aged mice. Data represents mean ± SEM, n=6 for each strain magnitude. Where it was possible to fit a two-stage linear regression line, this line is shown on the relevant graph. ***p<0.001: the gradient of the load-response regression line being different from zero. p<0.05: the overall difference between young and aged lines determined by regression analysis.

Supplementary Figure 4 The effect of loading on cortical bone in young and aged male and female mice. Percentage change [(right - left) / left] * 100 in medullary area (A), bone area fraction (B) and cortical thickness (C) were compared in young and aged mice. Data represents mean ± SEM, n=6 for each strain magnitude. Where it was possible to fit a two-stage linear regression line, this line is shown on the relevant graph. For medullary area in males, it was only possible to fit linear regression lines. *p<0.05, **p<0.01, ***p<0.001: the gradient of the load-response regression line being different from zero.

Supplementary Figure 5 The effect of loading and ageing on the proportion of cells in G_1_/S or S phase of the cell cycle. Proportion of Ki67 positive cells in G_1_/S- (A, B) and S-phase (C, D) of the cell cycle were calculated by analysis of Ki67 nuclear patterning using immunofluorescence. n=4 per group with 2 repeats in males and 3 repeats in females. Bars represent mean and SEM. **p<0.01 compared to aged static controls (by paired t-test).

Supplementary Table 1 Loading-engendered strains measured at the 37% site of the proximal tibia in representative mice were used to determine the magnitude of load required to engender strain magnitudes of 500, 1000, 1500, 1750, 2000, 2250 and 2500 on the medial surface of the tibia at the 37% site measured from the proximal end in young and aged, male and female mice. The load rate to apply an average strain rate of 30,000s^-1^ during loading and unloading was also calculated. Strain magnitudes are greater in the posterior-lateral region of the bone cortex where the magnitude is approximately 2.5 times higher^[9]^.
