# Supplementary figures and images for: Age-Related Impairment of Bones' Adaptive Response to Loading in Mice Is Associated With Sex-Related Deficiencies in Osteoblasts but No Change in Osteocytes
Source: J Bone Miner Res. 2014 Jul 21;29(8):1859–71. doi: 10.1002/jbmr.2222 (PMC4258100; doi:10.1002/jbmr.2222)

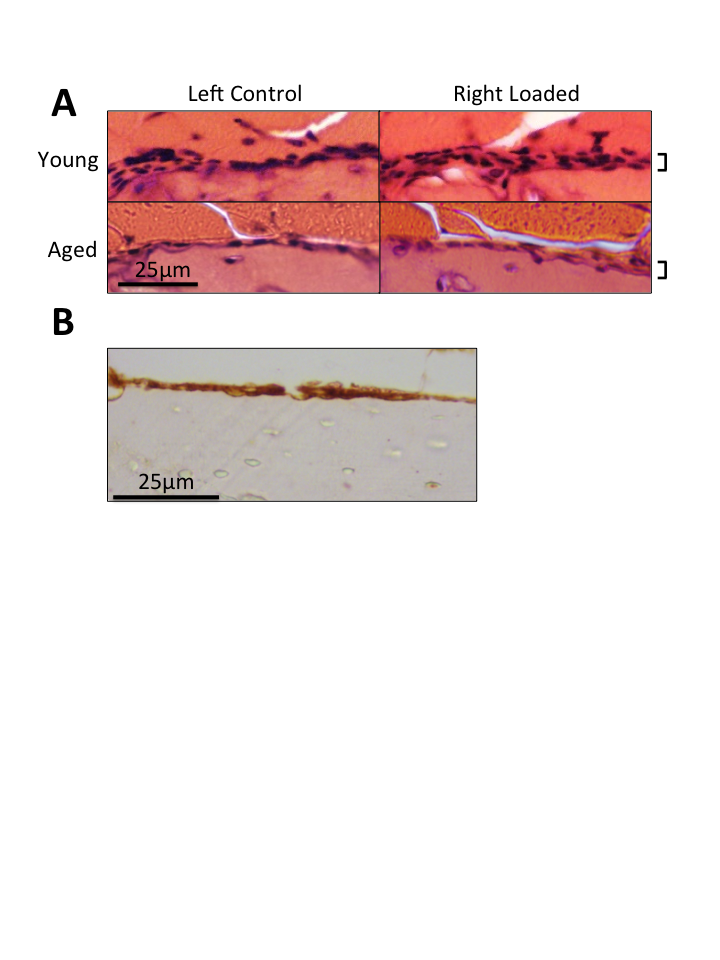

Supplement: Supplementary Figure 1 — Loading increased the number of osteoblastic cells in the periosteum of young but not old mice. (A) Representative H&E sections from the posterior-lateral (high strain) region of the tibia of male mice 24 hours after artificial loading. Cells were counted in the periosteum which is indicated by the open brackets. (B) Periostin staining was used to confirm that the cells counted were osteoblastic as illustrated in a representative high power image. Scale bar = 25m. [file jbmr0029-1859-SD1.tif]

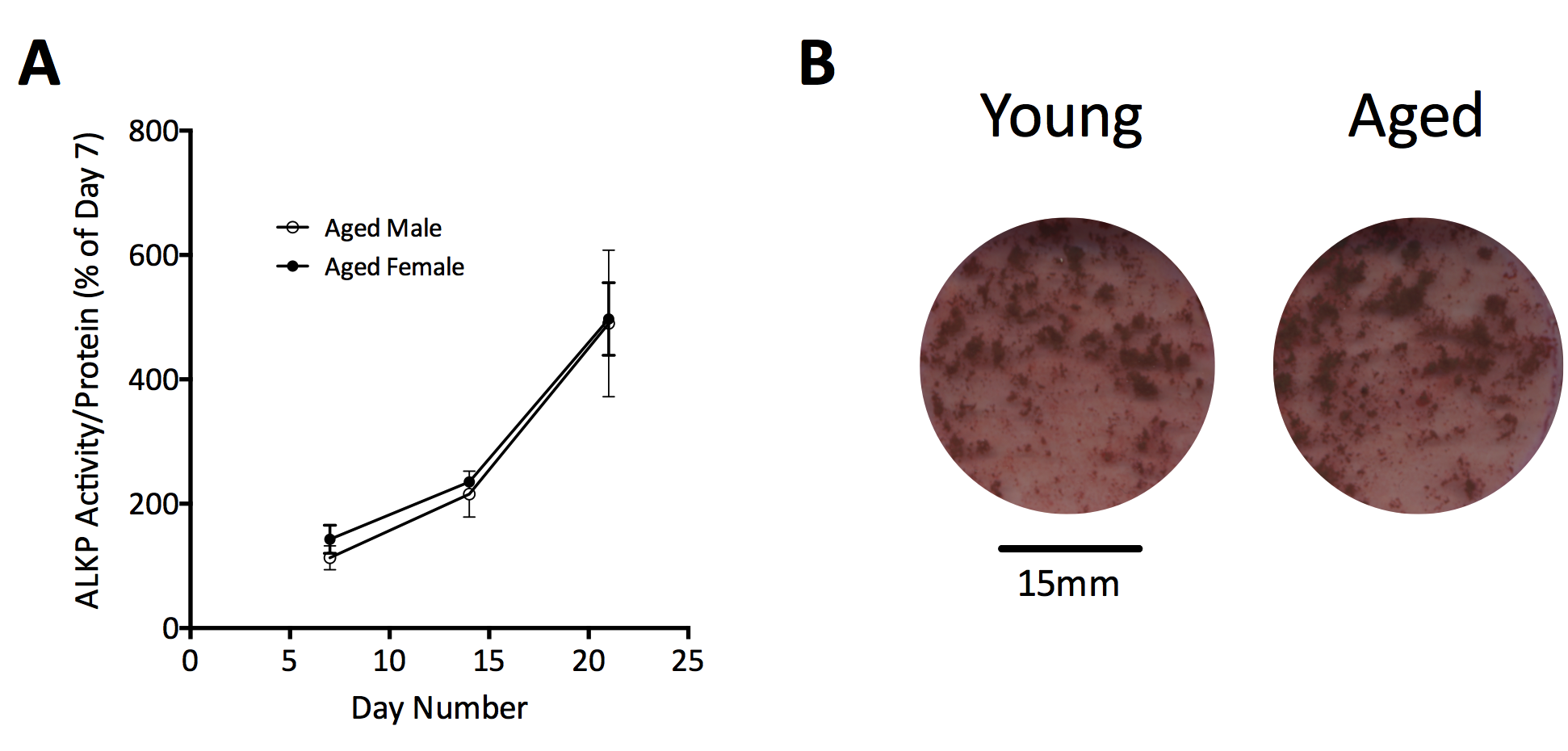

Supplement: Supplementary Figure 2 — Cells cultured from aged mice are osteoblastic in nature as shown by their expression of alkaline phosphatase and their ability to form mineralized nodules. (A) ALP activity was measured between 7 and 21 days in osteoblastic cells cultured from the long bones of aged male and female mice. Results are corrected for protein content and expressed as a percentage of ALP activity at day 7. (B) After 21 days in culture in complete medium containing 50µM ascorbic acid and 10mM β-glycerol phosphate, cells from young and aged mice formed mineralized nodules as shown by Alizarin Red staining. Scale bar = 15mm. [file jbmr0029-1859-SD2.tif]

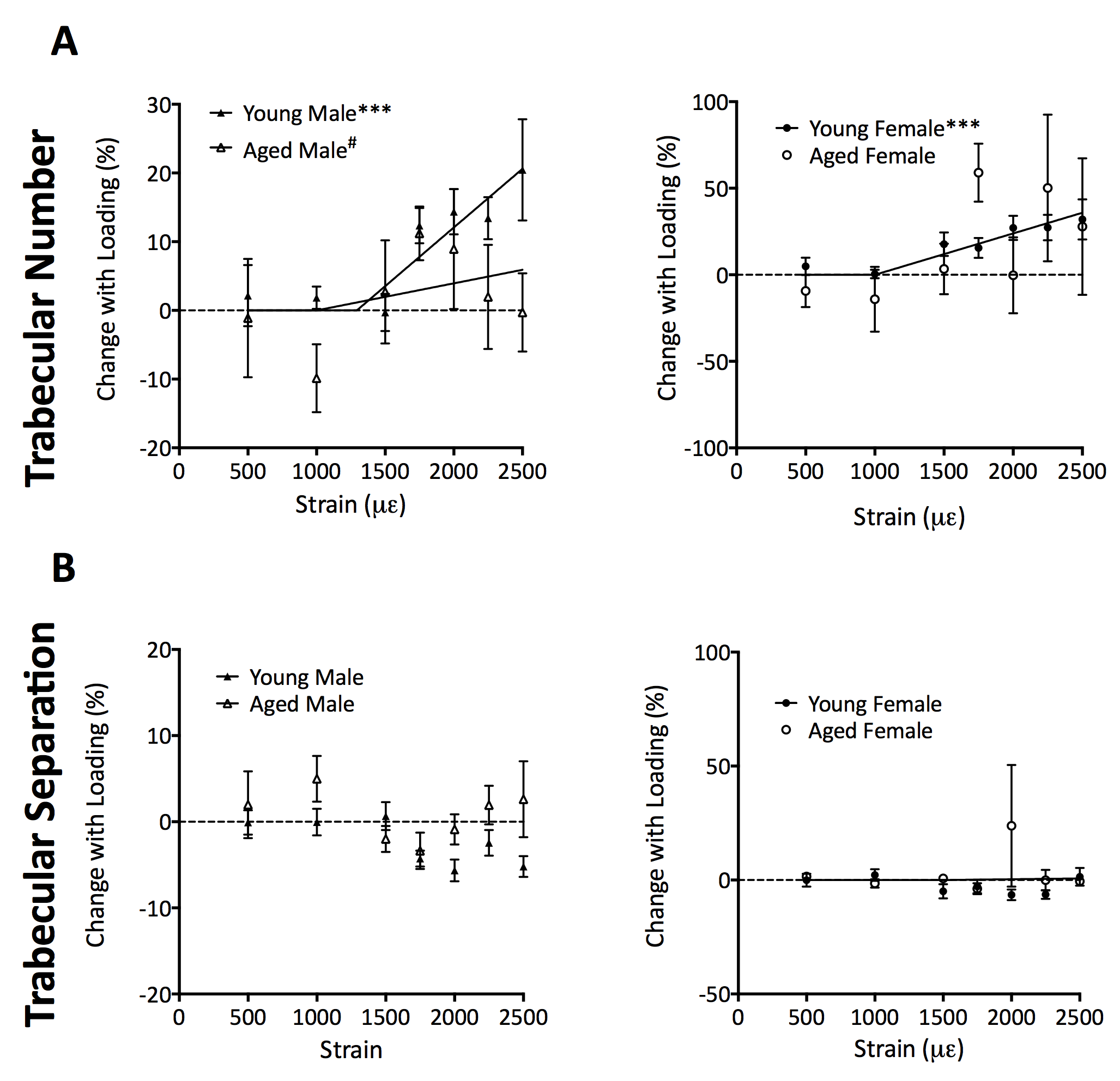

Supplement: Supplementary Figure 3 — The effect of loading on trabecular bone in young and aged male and female mice. Percentage change [(right - left) / left] * 100 in trabecular number (A) and separation (B) were compared in young and aged mice. Data represents mean ± SEM, n=6 for each strain magnitude. Where it was possible to fit a two-stage linear regression line, this line is shown on the relevant graph. ***p<0.001: the gradient of the load-response regression line being different from zero. p<0.05: the overall difference between young and aged lines determined by regression analysis. [file jbmr0029-1859-SD3.tif]

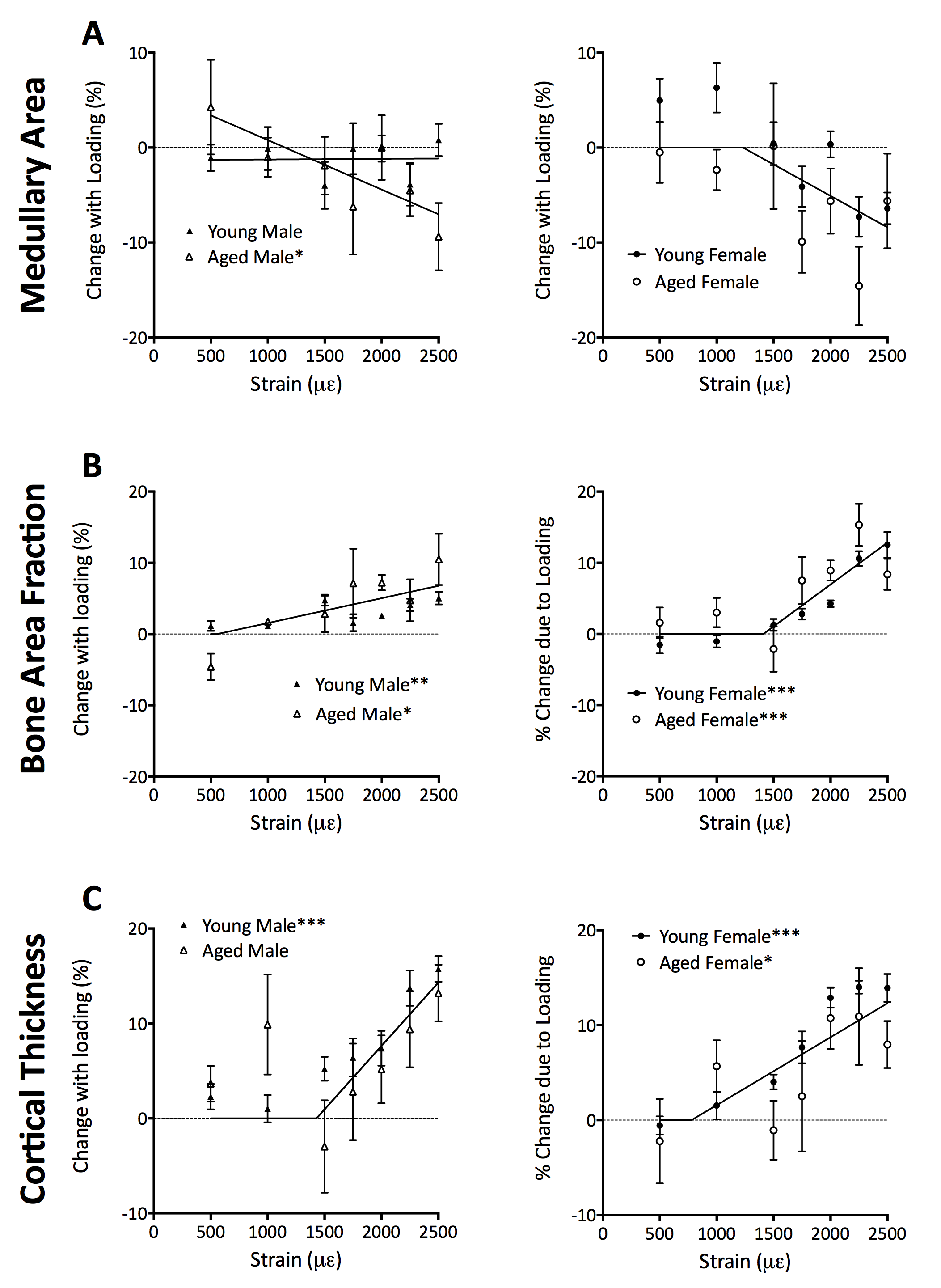

Supplement: Supplementary Figure 4 — The effect of loading on cortical bone in young and aged male and female mice. Percentage change [(right - left) / left] * 100 in medullary area (A), bone area fraction (B) and cortical thickness (C) were compared in young and aged mice. Data represents mean ± SEM, n=6 for each strain magnitude. Where it was possible to fit a two-stage linear regression line, this line is shown on the relevant graph. For medullary area in males, it was only possible to fit linear regression lines. *p<0.05, **p<0.01, ***p<0.001: the gradient of the load-response regression line being different from zero. [file jbmr0029-1859-SD4.tif]

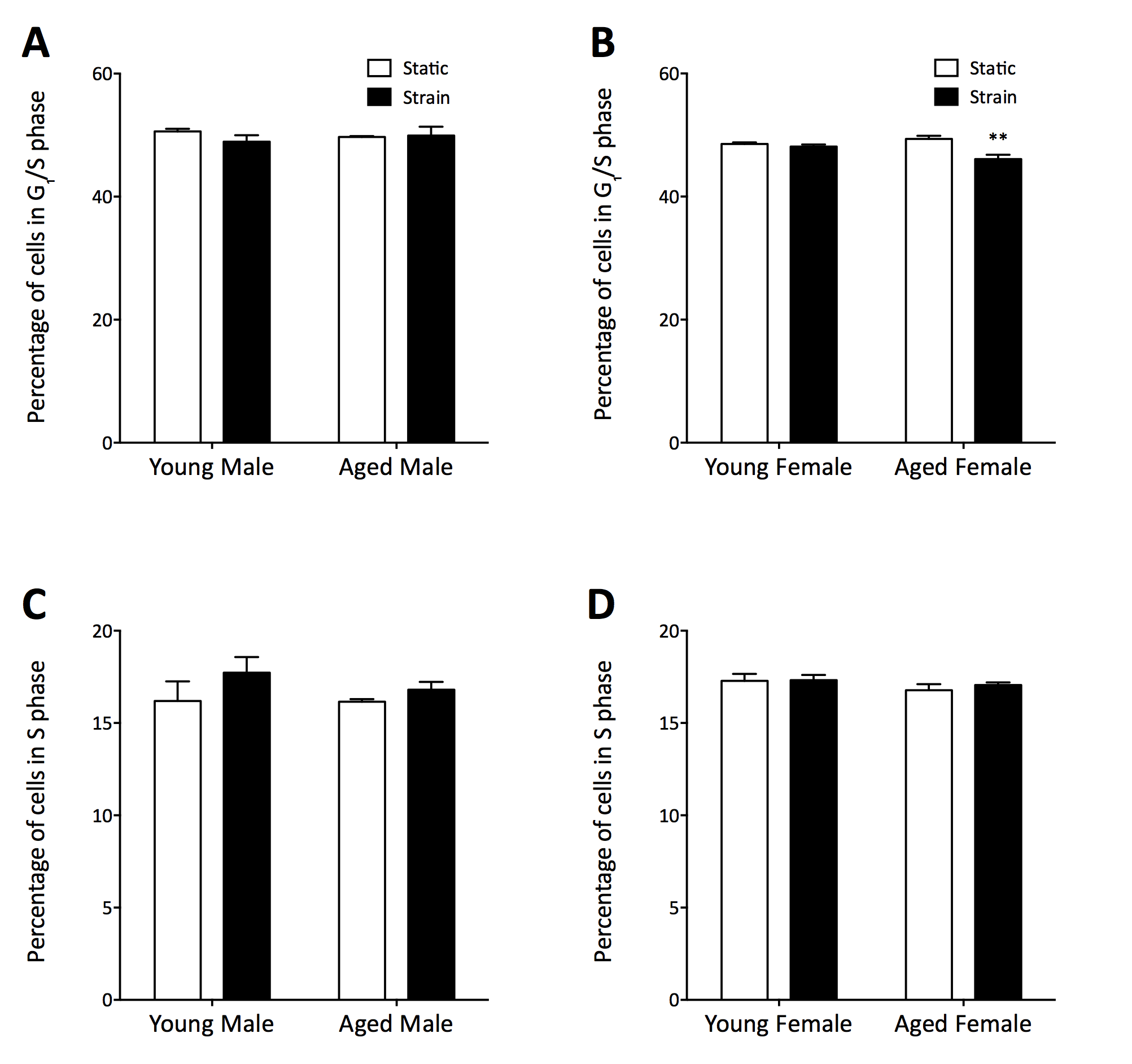

Supplement: Supplementary Figure 5 — The effect of loading and ageing on the proportion of cells in G1/S or S phase of the cell cycle. Proportion of Ki67 positive cells in G1/S- (A, B) and S-phase (C, D) of the cell cycle were calculated by analysis of Ki67 nuclear patterning using immunofluorescence. n=4 per group with 2 repeats in males and 3 repeats in females. Bars represent mean and SEM. **p<0.01 compared to aged static controls (by paired t-test). [file jbmr0029-1859-SD5.tif]
