# Supplementary material for: Age-Related Impairment of Bones' Adaptive Response to Loading in Mice Is Associated With Sex-Related Deficiencies in Osteoblasts but No Change in Osteocytes
Source: J Bone Miner Res. 2014 Jul 21;29(8):1859–71. doi: 10.1002/jbmr.2222 (PMC4258100; doi:10.1002/jbmr.2222)
Supplement: Supplementary Table 1 — Loading-engendered strains measured at the 37% site of the proximal tibia in representative mice were used to determine the magnitude of load required to engender strain magnitudes of 500, 1000, 1500, 1750, 2000, 2250 and 2500 on the medial surface of the tibia at the 37% site measured from the proximal end in young and aged, male and female mice. The load rate to apply an average strain rate of 30,000s-1 during loading and unloading was also calculated. Strain magnitudes are greater in the posterior-lateral region of the bone cortex where the magnitude is approximately 2.5 times higher[9]. [file jbmr0029-1859-SD6.doc]

Supplementary Table 1

| Strain (με) | Young Male | Aged Male | Young Female | Aged Female |
| --- | --- | --- | --- | --- |
| 500 | 3.06N | 2.23N | 2.99N | 2.35N |
| 1000 | 6.12N | 4.46N | 5.99N | 4.71N |
| 1500 | 9.18N | 6.69N | 8.98N | 7.06N |
| 1750 | 10.71N | 7.81N | 10.48N | 8.24N |
| 2000 | 12.25N | 8.92N | 11.97N | 9.41N |
| 2250 | 13.78N | 10.04N | 13.47N | 10.59N |
| 2500 | 15.31N | 11.15N | 14.97N | 11.77N |
| Load Rate | 511N/s | 372N/s | 500N/s | 393N/s |

Supplementary Table 1: Loading-engendered strains measured at the 37% site of the proximal tibia in representative mice were used to determine the magnitude of load required to engender strain magnitudes of 500, 1000, 1500, 1750, 2000, 2250 and 2500με on the medial surface of the tibia at the 37% site measured from the proximal end in young and aged, male and female mice. The load rate to apply an average strain rate of 30,000μεs^-1^ during loading and unloading was also calculated. Strain magnitudes are greater in the posterior-lateral region of the bone cortex where the magnitude is approximately 2.5 times higher^[9]^.
